# Impact of bariatric surgery on infertility in obese women: a systematic review and meta-analysis

**DOI:** 10.1097/MS9.0000000000002657

**Published:** 2024-10-23

**Authors:** Behnam R. Makhsosi, Pourya Ghobadi, Masoumeh Otaghi, Zeinab Tardeh

**Affiliations:** a Department of Laparoscopic Surgery, School of Medicine, Imam Reza Hospital, Kermanshah University of Medical Sciences, Kermanshah, Iran; b Department of General Surgery, School of Medicine, Imam Reza Hospital, Kermanshah University of Medical Sciences, Kermanshah, Iran; c Department of Nursing and Midwifery Faculty, Ilam University of Medical Sciences, Ilam, Iran

**Keywords:** bariatric surgery, infertility, meta-analysis, obesity

## Abstract

**Background:**

The prevalence of obesity is increasing worldwide, with several consequences, including reduced fertility in both men and women. One of the ways to reduce weight is bariatric surgery. The aim of this systematic review and meta-analysis study was to evaluate the effectiveness of bariatric surgery on weight loss and subsequent improvement of fertility in obese infertile women.

**Methods:**

Several databases were searched using MESH terms to investigate the studies that evaluated the fertility rates after bariatric surgery in infertile obese women. Related data were obtained and analyzed using Stata software with a *P*-value ≤0.05, which was considered as significant.

**Results:**

Of the 283 articles related to the purpose of this study, 9 articles, including 231 obese infertile women, were included in the study. BMI before surgery was 42.2 [95% CI= 39.2–45.2] and BMI after surgery was 31.9 (95% CI= 28.9–34.8), which shows the significance of weight loss after bariatric surgery. Conception rate after bariatric surgery weight loss was increased and was 67% (95% CI= 47–87%, *P*-value <0.05).

**Conclusions:**

Bariatric surgery had a significant effect on the reduction of BMI and subsequent significant improvement of fertility in obese infertile women.

## Introduction

Highlights

- The prevalence of obesity is one of the increasing problems.
- Most of obese people are women of reproductive age.
- Obesity disrupts reproductive processes.
- Bariatric surgery have a significant effect on reducing BMI.
- Bariatric surgery have a significant effect on improving fertility.

The prevalence of obesity is a widespread and growing problem^1^. The statistics of this problem as one of the health issues in 2016 reached 650 million people, which is 13% of the world’s population^2,3^. Approximately 80% of obese people are women, most of whom are of reproductive age^3^. Studies show a link between obesity and infertility, suggesting that fertility increases with weight loss in obese women^1^. Obesity, as one of the risk factors during pregnancy, has adverse consequences for both mother and fetus, including difficult pregnancy, miscarriage, and congenital abnormalities^4^. Obesity disrupts reproductive processes and characteristics by disrupting the normal hormone levels^5^. Therefore, weight loss in obese women regulates menstrual and ovulatory cycles and increases the likelihood of fertility^6^. Drug treatment for infertility is usually not prescribed because of the side effects of some medications^7^. Despite the conflicting results of some studies on the positive effect of weight loss on improving fertility^8,9^, especially through surgery, most studies confirm this relationship^10^. In obese patients who have failed drug treatment, surgery should be considered as an obesity treatment^1^. Bariatric surgery is usually considered in people with severe obesity who have not been treated by other methods, such as medical therapy and lifestyle changes^11^. The most effective long-term weight loss treatment is bariatric surgery. It should be noted that more than half of these surgeries are performed on women who are of reproductive age^12^. Since its introduction (1960s), bariatric surgery has been gradually become accepted and has become the main method of obesity treatment^11^. Bariatric surgery includes a group of surgeries on the stomach or intestines^11^. This type of surgery, as the most effective treatment method for morbid obesity, is divided into two categories: malabsorption and restriction^5^. The most commonly used surgical procedures are already Roux-en-Y gastric bypass (RYGB), sleeve gastrectomy (SG), and adjustable gastric banding(AGB)^11^. SG, gastric bypass, and laparoscopic AGB are some of the most commonly used procedures in the world^5^.

According to previous studies bariatric surgery can improve fertility but the rate of increase in fertility after bariatric surgery in obese infertile women has not been determined by a comprehensive study. This study was conducted to examine the complications of obesity in infertile women and the beneficial effects of bariatric surgery on weight loss and fertility.

## Method

### Study protocol

This study was conducted using PRISMA (Preferred Reports for Systematic Reviews and Meta-Analyses) to determine the effect of bariatric surgery on infertility in obese women.

### Search strategy

The databases Web of Science (ISI), PubMed, Cochrane Library, Wiley Online Library, CINAHL, EMBASE, Scopus, Springer, Science Direct, and Google Scholar search engines were searched without a time limit to find the relevant studies. The MeSH keywords used included bariatric surgery, obesity, Roux-en-Y gastric bypass, sleeve gastrectomy, adjustable gastric banding, one anastomosis gastric bypass (OAGB), and infertility. These steps were performed and reviewed by two of the authors.

### Inclusion and exclusion criteria

Inclusion criteria were studies evaluated the changes in fertility rate after bariatric surgery. This review excluded nonhuman and non-English studies, as well as case reports, case collections, letters, articles with insufficient data, studies did not show the changes in the rate of conception after surgery and cases for which full text was not available.

### Quality assessment

In this phase of the study, all collected studies were simultaneously and independently assessed by two authors using the modified Newcastle–Ottawa Scale (NOS), and finally, those articles that received an acceptable score of 6 or more were included in the study.

### Study selection

To perform an initial screening to select relevant articles, endnote software was used to store and remove duplicate articles. The titles and abstracts of the articles were then scanned. The next step was to examine the full text of the articles.

### Data extraction

Based on the information extraction table, the data was extracted by the researchers. The extracted data included the following: author name and year of article publication, country name, and study type, number of infertile women, mean age, presurgery BMI, postsurgery BMI, follow-up, (month), live birth rate (LBR), conception, and type of surgery. These data were then stored using an Excel program.

### Statistical analysis

In this study, descriptive statistics (mean, median, and SD for continuous variables and frequency and percentage for categorical variables) were used. A weighted mean was used to estimate the pooled mean and 95% CI of BMI. The Metaprop command in Stata software was used to determine the incidence of conception among infertile women. Random-effects meta-analysis was used in this study. Heterogeneity among included studies was assessed using *I* ^2^ and *Q* statistics. A sensitivity analysis was performed to determine the robustness of the results. At each stage, one study was omitted and the results were compared with the full analysis. Stata software (version 14.2) was used for all statistical analyses. In addition, a funnel plot was used to assess publication bias. In addition, all comparisons were two-tailed with a threshold *P*-value ≤0.05 for statistical significance.

## Results

### Study characteristics and methodological quality

**Figure 1:**
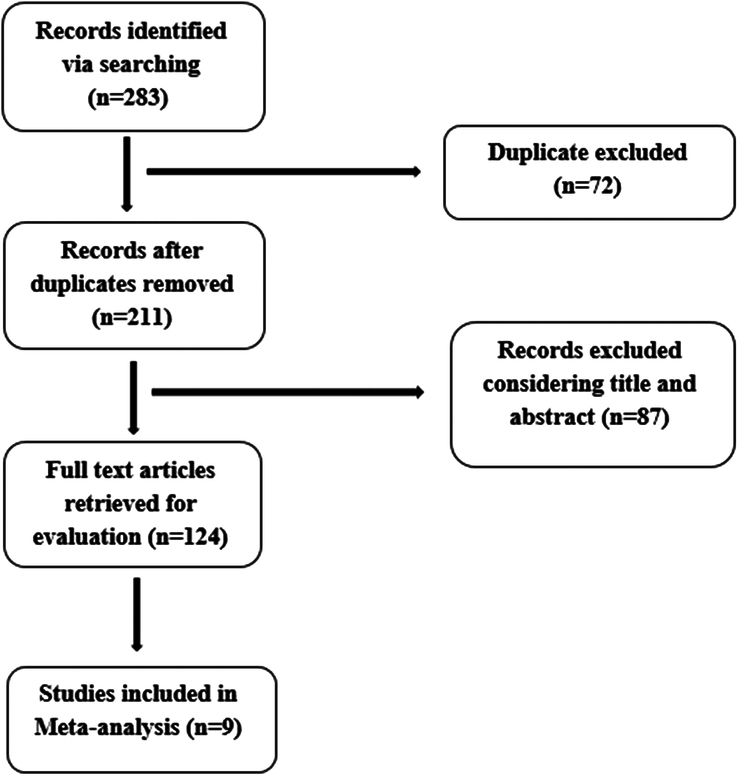
PRISMA flowchart.

After a complete review of the collected articles and removing 72 duplicates items and reviewing 87 abstracts and 124 complete articles finally, of the 283 articles collected, 9 articles including 231 infertile obese women were included in the meta-analysis (Fig. 1, and Table 1).

**Table 1:** Data obtained from a review of studies on the impact of bariatric surgery on infertility.

Author, year (reference)	Country	Number of infertile women	Mean age (SD)	BMI before surgery	BMI after surgery	Follow-up (month)	Conception	LBR	Time to pregnancy (month)	Type of surgery
Jamal, 2012^13^	US	6 PCOS	–	–	–	46.7	6	Six (5 spontaneous + 1 IUI)	Within 3 years	RYGB
Ahmed, 2017^14^	Iraq	16 (8 PCOS and 8 non PCOS)	30.2 (4.04)	46 (7.8)	35.3 (4)	48	14	14 (spontaneous pregnancy)	3.4 (0.3)	LSG
Milone, 2017^15^	Italy	40 with a history of ART failure	31.4 (4.7)	40.7 (2)	35 (2.6)		15	14 ART	–	LSG
Khazraei, 2017^16^	Iran	15	–	–	–	20	7	Five (spontaneous)	–	LSG
Menke, 2019^17^	US	52 (27 PCOS and 25 non PCOS)	–	–	–	78	22	21 (spontaneous pregnancy)	–	33 RYGB and 16 LAB and 3 other
Nayak, 2020^18^	India	28 (17 PCOS & 11 non PCOS)	29.56 (4.2)	42 (4.6)	30.14(3.9)	12	18	18 (14 spontaneous, 4 ICSI)	–	LSG
Öner, 2023^4^	Turkey	23 (PCOS and non PCOS)	31.26 (5.06)	45.04(3.4)	28.6 (3.1)	12	21	15 (10 spontaneous, 3 IVF, 2 S+IVF)	25.6 (4.7)	LSG
Abbas, 2023^19^	Pakistan	56 PCOS	30.8 (4.2)	45.2 (5.6)	27.8 (4.2)	34	53	47 (spontaneous)	34 (26)	Not sustained

### The effect of bariatric surgery on obese women BMI

**Figure 2:**
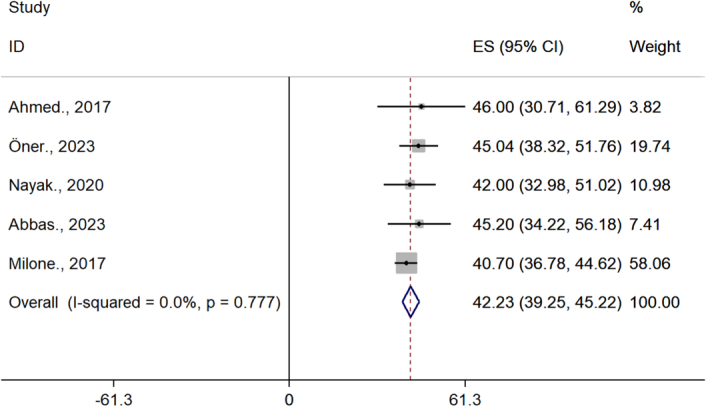
BMI of infertile patients before bariatric surgery.

**Figure 3:**
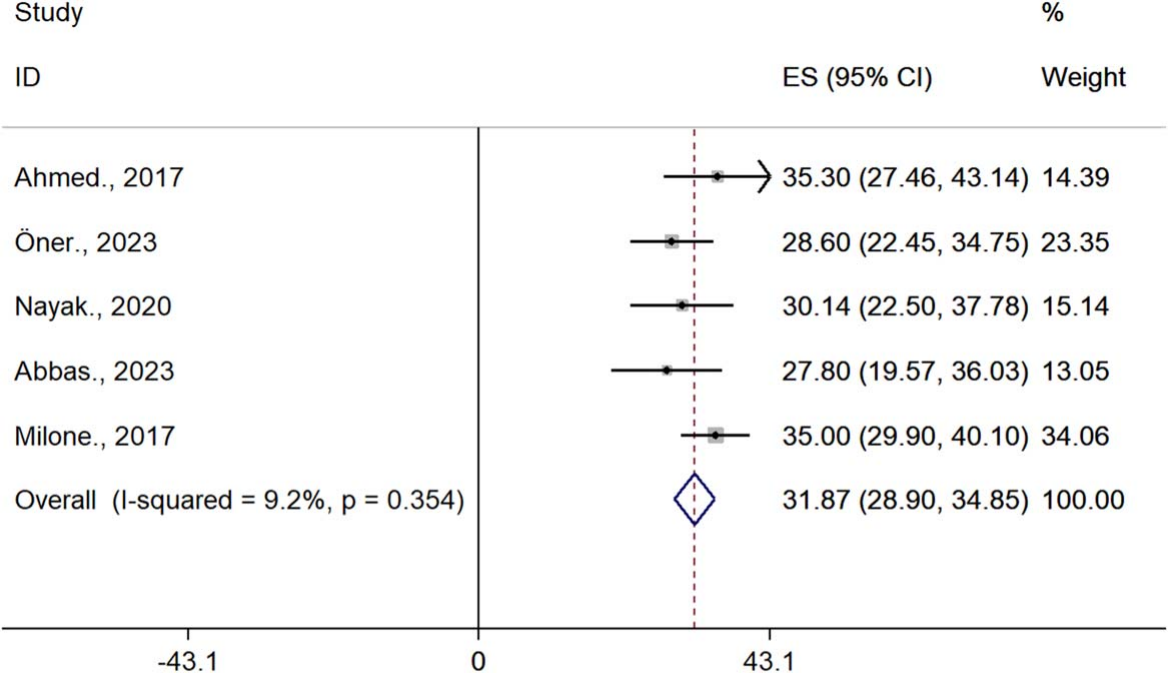
BMI of infertile patients after bariatric surgery.

The BMI of infertile patients before and after surgery was 42.2 (95% CI= 39.2–45.2) and 31.9 (95% CI= 28.9–34.8), respectively (Figs. 2, 3).

The analysis shows that there is a significant difference in the BMI of patients before and after surgery.

### The effect of bariatric surgery on infertility and subgroup analysis

**Figure 4:**
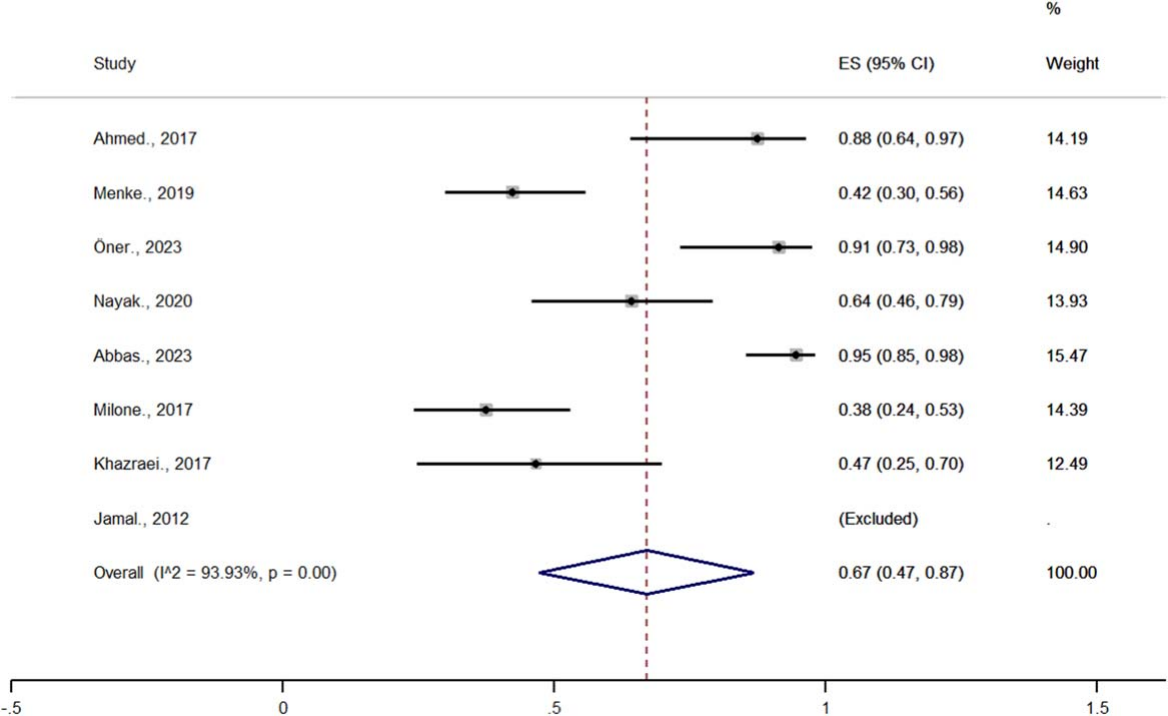
Meta-analysis of studies on the impact of bariatric surgery on infertility.

The overall pooled estimate of fertility after bariatric surgery was 67% (95% CI= 47–87%, *P*-value <0.05) (Fig. 4).

**Figure 5:**
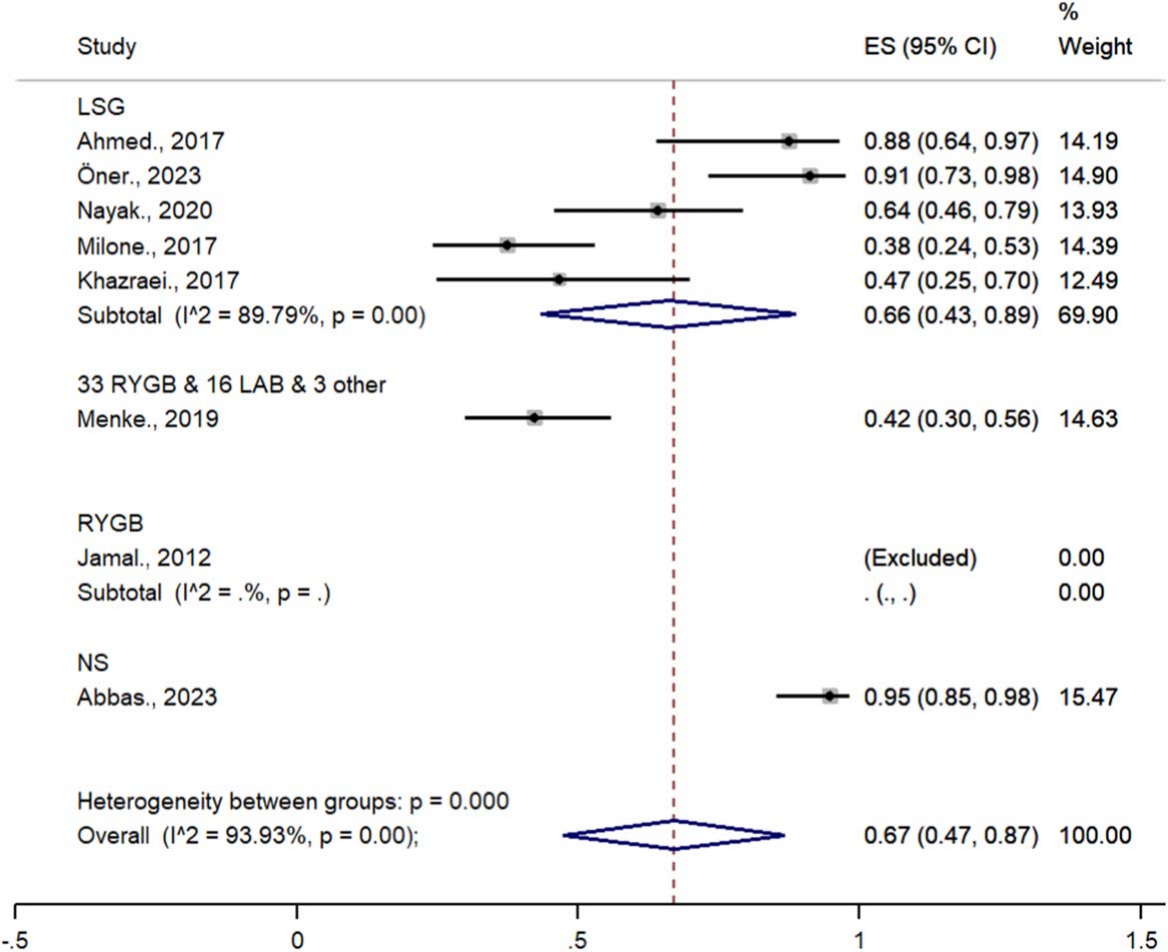
Subgroup analysis in a meta-analysis of studies on the impact of bariatric surgery on infertility.

Heterogeneity was high in this meta-analysis (*I* ^2^=93.9%; *P*-value=0.00), and the subgroup analysis was checked according to the type of surgery (Fig. 5). Due to the limitations of the studies and the lack of common data in this area, it was not possible to accurately determine the cause of heterogeneity using a subgroup of analysis. The time of conception from surgery was different in the included articles from 12 to 78 months. The average time to conception was 35.8 months.

### Sensitivity analysis

**Figure 6:**
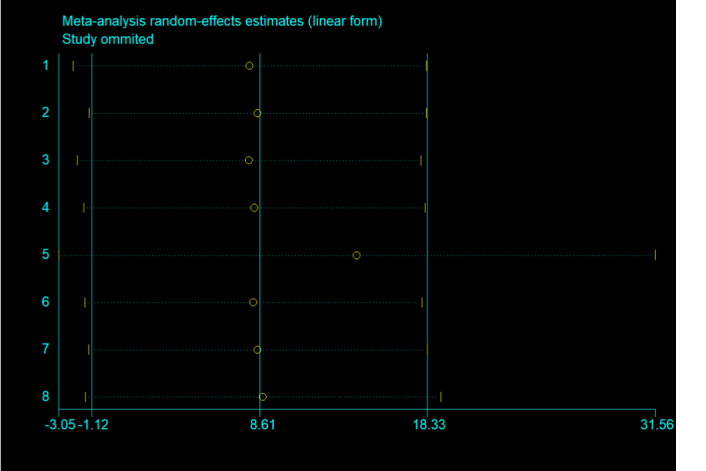
Sensitivity analysis in a meta-analysis of studies on the impact of bariatric surgery on infertility.

For sensitivity analysis, reanalysis was performed after excluding each study in the remaining studies, and according to the evaluation, our findings were not affected by the excluded studies Fig. 6.

**Figure 7:**
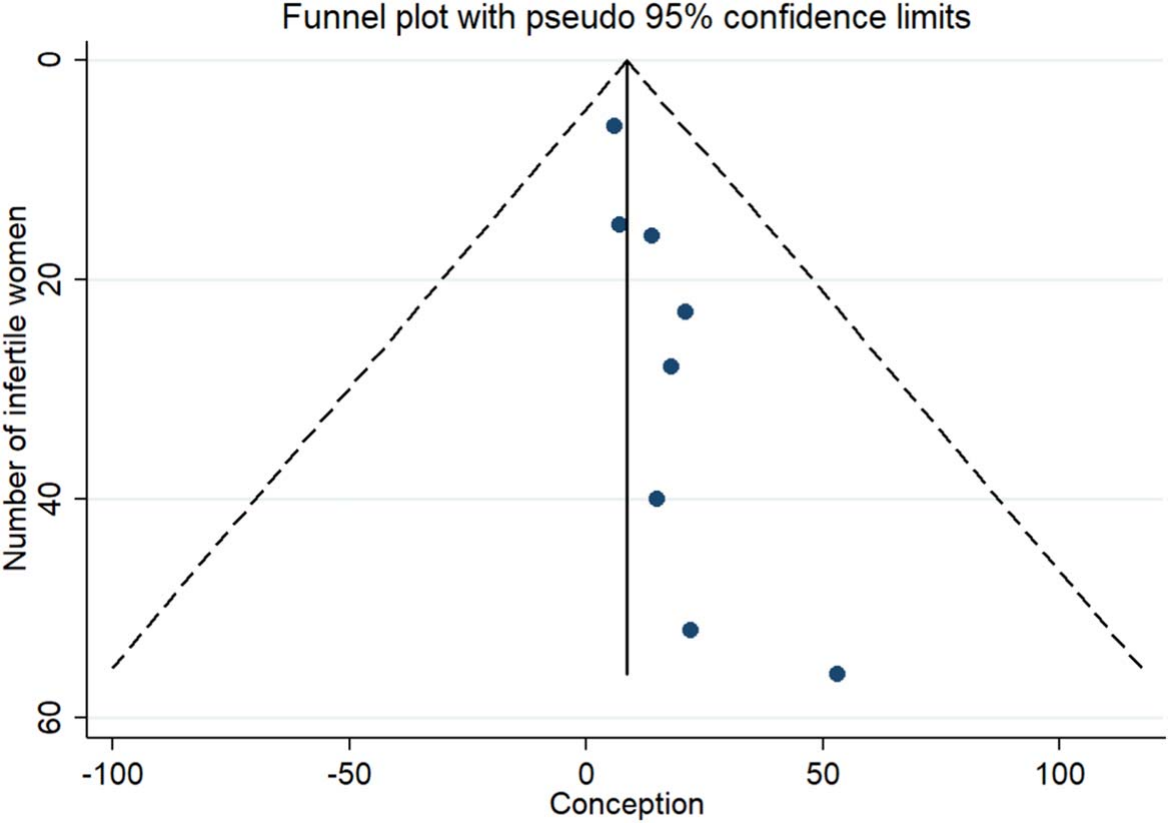
Publication bias in a meta-analysis the impact of the bariatric surgery on infertility.

### Publication bias

According to Egger’s test and funnel design, no bias was found among the articles analyzed (*P*>0.05) Fig. 7.

## Discussion

According to the results of the meta-analysis, there is a direct relationship between obesity and infertility. In addition to obesity, PCOS is one of the major factors that increase the likelihood of infertility in overweight women. The causes of the effect of obesity on infertility have been explained. For example, the fat cells of obese women produce a lot of leptin, which can alter the menstrual cycle by disrupting the hormonal balance and lead to infertility^5^. Therefore, the relationship between obesity and infertility is strong, and weight loss plays an effective role in the treatment of infertility in obese women^1^. Bariatric surgery has been shown to play an effective role in the treatment of infertility^20^. One of the most effective ways to treat obesity is bariatric surgery, which improves libido and sexual activity by improving PCOS symptoms (hormonal changes, lack of ovulation, and hirsutism)^4^. According to the analysis of nine articles reviewed in this study, bariatric surgery leads to weight loss and improvement of infertility in women by 67%. In the study conducted by Christinajois *et al*.^13^ to investigate LSG in obese women (40 people), the results showed that the fertility rate after surgery was 43.75%.

The results of a meta-analysis study showed the positive effect of bariatric surgery on women with PCOS and obesity, with a significant reduction in the prevalence of PCOS, menstrual irregularity, and hirsutism 1 year after surgery^14^. In another study, it was reported that the percentage of fertility in infertile obese women with PCOS reached 100% (six patients) after bariatric surgery^15^. BMI delays conception in a dose-dependent manner in women^16,17^. Studies have shown that high BMI can cause ovulation failure, followed by menstrual irregularity and impaired oocyte development, and its direct effect on the endometrium has been demonstrated^18^. Several studies report that reducing BMI after bariatric surgery plays an effective role in reducing the risk of delivering a macrosomic baby^19^. Musella and colleagues (2012) found that BMI and degree of weight loss after surgery were notable predictors of pregnancy^1^. These results are supported by the present study.

Qurashi *et al*. evaluated the effects of bariatric surgery on both genders and demonstrated improvement in erectile function scores, increased total and free testosterone, decreased estradiol and increased sperm morphology in males. They demonstrated an increase in sex hormone levels (FSH, LH, and SHBG) and sexual function in both sexes, and decreased total and free testosterone and estradiol in females, but menstrual irregularities, total sperm count, semen volume, sperm motility, and concentration did not significantly changed at 12 months follow-up^21^. According to previous studies, bariatric surgery improves the rate of fertility in women by modulating the hypothalamic–pituitary–ovarian (HPO) axis with changes in ovarian hormone levels such as estradiol and progesterone. As BMI decreases after bariatric surgery levels of leptin and two adipokines, leptin and adiponectin will change that may affect the fertility^22^. Studies have also shown another potential reason of increase in fertility after bariatric surgery is increase in antimullerian hormone (AMH) levels by weight loss in patients with and without PCOS^23^. Another studies support that after bariatric surgery and BMI loss, sexual function will improve in women who do not have pelvic floor disorders^24^. Although bariatric surgery can increase fertility, but time-to-conception interval must be considered and special care is needed before, during and after pregnancy^11,25^. Despite using supplementations the rate of micronutrient deficiency in post bariatric surgery pregnant women is high that shows the need for proper monitoring of nutrition by a multidisciplinary team to maintain the health of mother and fetus^26^. Potential effects of bariatric surgery on pregnancy is reducing the risk of preeclampsia, gestational diabetes or impaired fasting glucose and large-for-gestational-age neonates but increases the risk of small-for-gestational-age (SGA) neonates^27^. The rates of gestational diabetes mellitus, gestational hypertension, and cesarean section can reduce significantly after bariatric surgeries^28^. Considering the present meta-analysis, the heterogeneity was high, but there were few similar articles, the source of heterogeneity was unclear, and the only subgroup investigated was the type of surgery, the result of this subgroup is not valuable due to the limitation of the studies in each subgroup and more comprehensive studies are needed to find the procedure of choice. Biases present in the included studies or discrepancies in study designs is the reason of limitation of the studies in each subgroup.

## Conclusion

Bariatric surgery, by reducing the weight of obese patients, effectively leads to restoration of the menstrual cycle and improvement of hyperandrogenism as well as its clinical manifestations and subsequently causes ovulation and normal pregnancy in pregnant patients. Therefore, bariatric surgery may be an appropriate treatment option for obese infertile patients, especially if they have not been treated with medications and lifestyle changes. Further studies are recommended.

## Ethical approval

Kermanshah University of Medical Sciences Research Committee.

## Consent

This is a meta-analysis study and we did not have access to patients.

## Source of funding

There related data was declared.

## Author contribution

Z.T., P.G.H., and M.O.: acquired the data; Z.T.: analyzed and interpreted data; B.M., Z.T., and P.G.H.: drafted the manuscript; B.M. and M.O.: critically revised the manuscript; B.M.: supervised the study. All authors have read and approved the manuscript.

## Conflicts of interest disclosure

The authors declare no conflicts of interest.

## Guarantor

Zeinab Tardeh.

## Data availability statement

Datasets generated during and analyzed during the current study are publicly available.

## Provenance and peer review

The paper is not commissioned, externally peer-reviewed.
